# The decision to germinate is regulated by divergent molecular networks in spores and seeds

**DOI:** 10.1111/nph.14018

**Published:** 2016-06-03

**Authors:** Eleanor F. Vesty, Younousse Saidi, Laura A. Moody, Daniel Holloway, Amy Whitbread, Sarah Needs, Anushree Choudhary, Bethany Burns, Daniel McLeod, Susan J. Bradshaw, Hansol Bae, Brian Christopher King, George W. Bassel, Henrik Toft Simonsen, Juliet C. Coates

**Affiliations:** ^1^ School of BiosciencesUniversity of BirminghamEdgbastonBirminghamB15 2TTUK; ^2^ Department of Systems BiologyTechnical University of DenmarkSøltofts Plads, 2800 KgsLyngbyDenmark; ^3^ Department of Plant and Environmental SciencesUniversity of CopenhagenThorvaldsensvej 40Frederiksberg C1871Denmark

**Keywords:** abscisic acid (ABA), *ent*‐kaurenes, ethylene, high temperature, light, *Physcomitrella*, spore germination, strigolactones

## Abstract

- Dispersal is a key step in land plant life cycles, usually via formation of spores or seeds. Regulation of spore‐ or seed‐germination allows control over the timing of transition from one generation to the next, enabling plant dispersal. A combination of environmental and genetic factors determines when seed germination occurs. Endogenous hormones mediate this decision in response to the environment. Less is known about how spore germination is controlled in earlier‐evolving nonseed plants.
- Here, we present an in‐depth analysis of the environmental and hormonal regulation of spore germination in the model bryophyte *Physcomitrella patens* (*Aphanoregma patens*).
- Our data suggest that the environmental signals regulating germination are conserved, but also that downstream hormone integration pathways mediating these responses in seeds were acquired after the evolution of the bryophyte lineage. Moreover, the role of abscisic acid and diterpenes (gibberellins) in germination assumed much greater importance as land plant evolution progressed.
- We conclude that the endogenous hormone signalling networks mediating germination in response to the environment may have evolved independently in spores and seeds. This paves the way for future research about how the mechanisms of plant dispersal on land evolved.

## Introduction

Transition from one generation to the next in land plants is mediated by the formation of desiccation‐resistant dispersal units (Finch‐Savage & Leubner‐Metzger, 2006). Within the spermatophyte lineage, these dispersal units are multicellular seeds, whereas in bryophytes and nonseed vascular plants (lycophytes and ferns) the functionally equivalent dispersal units are unicellular spores (Linkies *et al*., 2010). How and when germination is initiated in a seed or spore is critical for plant and species reproduction, movement and survival.

Hormonal and environmental factors are both well‐established as key players in the regulation of seed germination (Holdsworth *et al*., 2008a). The regulation of seed germination is highly complex, and involves integration of environmental signals by hormones within the seed and within different seed compartments and cell types (Yamaguchi *et al*., 2001; Holdsworth *et al*., 2008b; Linkies *et al*., 2010; Dekkers *et al*., 2013). Much is still unknown about exactly how seed germination is controlled at a cellular level (Nonogaki *et al*., 2010; Bassel *et al*., 2014).

Spores, unlike seeds, are haploid and are derived from the sporophyte stage of the plant life cycle via meiosis (reviewed in Rubinstein *et al*., 2010). Despite the different developmental origins of spores and seeds, previous work suggests that at least some aspects of germination regulation may be conserved between the two types of dispersal unit, as outlined later. Because spores are unicellular structures, understanding spore germination provides us with a simplified system for the study of the cellular and hormonal basis of germination regulation, and how this has evolved.

Environmental regulation of seed germination in both monocots and dicots is controlled in part by a phytochrome‐mediated reversible system, with red (R) light promoting germination, and even brief exposure to far‐red (FR) light inhibiting R light‐induced germination (Borthwick *et al*., 1952; Shinomura *et al*., 1994; Hennig *et al*., 2002), although this trait has been bred out of some commercial cereal crops (Barrero *et al*., 2012). A similar R–FR reversible system regulates spore germination in several ferns (Mohr *et al*., 1964; Raghavan, 1973; Wayne & Hepler, 1984; Scheuerlein *et al*., 1989; Tsuboi *et al*., 2012). In the earliest‐evolving land plant lineage, bryophytes, complete inhibition of spore germination by FR light, and reversal of this inhibition by R light via phytochromes, has been demonstrated (Possart & Hiltbrunner, 2013). Phytochrome regulation of spore germination is likely to be extremely ancient, as it appears to exist outside the land plant lineage, also, including in spores of the Charophycean algae *Spirogyra* and *Chara*, and in fungi (Calpouzos & Chang, 1971; Takatori & Imahori, 1971; Lucas *et al*., 1975; Mathews, 2006; Agrawal, 2009). The mechanisms downstream of phytochromes that regulate the control of germination throughout the plant lineage are poorly understood.

The onset of germination in seeds is closely regulated by the balance between plant hormone signalling pathways of gibberellin (GA) and abscisic acid (ABA), which interact at multiple levels (Karssen & Lacka, 1986; Holdsworth *et al*., 2008a). We have recently shown that ABA inhibits spore germination in *Physcomitrella* and that conserved proteins modulate ABA‐mediated germination responses in both spores and seeds (Moody *et al*., 2016). This suggests that downstream signalling components regulating germination may be conserved between spores and angiosperm seeds. In seeds, gibberellins are required for germination: seeds of the *Arabidopsis ga1* mutant, which lacks the first enzyme in the GA biosynthesis pathway, are unable to germinate without exogenously supplied gibberellin (Koornneef & van der Veen, 1980), whereas GA receptor (GID) mutants cannot germinate fully (Voegele *et al*., 2011). GA overcomes the inhibitory effects of ABA to allow seed germination (Holdsworth *et al*., 2008a).

Conflicting results relating to the role of GA and ABA in the control of plant spore germination have been reported. Mosses biosynthesize the diterpenes at the start of the GA biosynthesis pathway, *ent*‐kaurene and *ent*‐kaurenoic acid, but they lack the enzyme that further converts *ent*‐kaurenoic acid into bioactive gibberellins (as occurs in seed plants). Thus, the identity of bioactive diterpenes in spore‐bearing plants is not yet fully characterized (Von Schwartzenberg *et al*., 2004; Hayashi *et al*., 2010; Zhan *et al*., 2015). Microarray analysis of spore germination in the fern *Ceratopteris* implicated involvement of GA signalling and downregulation of ABA signalling in this process, similarly to seeds (Yao *et al*., 2008). However, different fern species' spores have different sensitivities to GA and ABA application (Weinberg & Voeller, 1969; Chia & Raghavan, 1982; Singh *et al*., 1990; Kagawa & Michizo, 1991; Haas *et al*., 1992). The GA biosynthesis inhibitor AMO‐1618, which blocks the first step(s) in the GA biosynthesis pathway (Rademacher, 2000), can inhibit some (but not all) light‐induced fern spore germination (Weinberg & Voeller, 1969; Nester & Coolbaugh, 1986; Kagawa & Michizo, 1991). In the best‐studied model bryophyte, the moss *Physcomitrella patens* (*Aphanoregma patens*), a *copalyl‐diphosphate synthase/kaurene synthase* (*cps/ks*) mutant, which lacks the CYP88A enzyme that catalyses the key step of *ent*‐kaurenoic acid oxidation in gibberellin biosynthesis and hence makes no diterpenes, had no reported spore germination phenotype (Hayashi *et al*., 2010). However, *Physcomitrella* spore germination can be inhibited by AMO‐1618, although AMO‐1618 may have targets in addition to the CPS enzyme (Anterola *et al*., 2009). Reports in other bryophytes detail the conflicting effects of exogenously applied gibberellins on spores of different species at different concentrations (Chopra & Kumra, 1988).

ABA is central to dormancy establishment and maintenance in *Arabidopsis* (Finkelstein *et al*., 2008). Freshly harvested *Arabidopsis* seeds contain high levels of ABA and show primary dormancy. ABA is also implicated in the imposition of secondary dormancy by, for example, high temperatures, via *de novo* synthesis of this hormone (Finch‐Savage & Leubner‐Metzger, 2006; Toh *et al*., 2008, 2012). The majority of bryophyte species’ spores have not been reported to show primary dormancy (McLetchie, 1999; Glime, 2015). Whether spores can have secondary dormancy imposed on them is currently not well‐characterized (Glime, 2015).

ABA also protects plants against abiotic stresses such as desiccation and freezing (Lee & Luan, 2012; Dekkers *et al*., 2015). Formation of both mature seeds and spores involves desiccation, and aspects of ABA signalling during abiotic stress responses in bryophytes are conserved with angiosperms (Knight *et al*., 1995; Cuming *et al*., 2007; Khandelwal *et al*., 2010). ABA‐mediated stress tolerance in bryophytes occurs at least in part via accumulation of soluble sugars including sucrose (Burch & Wilkinson, 2002; Nagao *et al*., 2006; Oldenhof *et al*., 2006; Bhyan *et al*., 2012; Erxleben *et al*., 2012). Notably, soluble sugars (sucrose, glucose) can inhibit germination in *Arabidopsis* seeds (Dekkers *et al*., 2004; Li *et al*., 2012).

Several additional hormones regulate seed germination. Strigolactones (SLs) produced by host plants are potent promoters of parasitic plant seed germination (Bouwmeester *et al*., 2003; Yoneyama *et al*., 2010). In *Arabidopsis*, strigolactone signalling pathway mutants show reduced seed germination (Stanga *et al*., 2013) and strigolactone can overcome the secondary dormancy imposed by exposing *Arabidopsis* seeds to high temperatures (Toh *et al*., 2012). Ethylene also promotes seed germination via multiple routes (Stewart & Freebairn, 1969; Logan & Stewart, 1991; Linkies & Leubner‐Metzger, 2012), whereas an inhibitory role for cytokinin in germination is implied in *Arabidopsis* (Riefler *et al*., 2006). A role for auxin in seed germination under normal conditions has not been demonstrated (Holdsworth *et al*., 2008a; Park *et al*., 2011); neither has the effect of these hormones on moss spore germination been investigated systematically (Chopra & Kumra, 1988; Glime, 2015).

Here, we sought for the first time to define comprehensively how hormones and environmental processes regulate spore germination in the bryophyte lineage by using *Physcomitrella* as a model system.

## Materials and Methods

### *Physcomitrella* culture and spore generation

*Physcomitrella patens* (Gransden wild‐type (WT) strain, and *Ppcps/ks* and *ccd8* mutants) was cultured and sporulation induced as in Moody *et al*. (2012). Sporophytes were harvested after maturation (dark brown sporophytes with a slightly ‘sparkly’ appearance) using sterile forceps under a SMZ645 dissecting microscope (Nikon, Tokyo, Japan) and air‐dried in sterile tubes for *c*. 1 wk before storage at room temperature.

### Spore germination assays

Spores from a minimum of three sporophytes (all of the same age) of a particular genotype were used within each assay. For larger assays, three sporophytes’ worth of spores were used for every 10 Petri dishes (9 cm diameter). For assays comparing WT with a mutant, spores of each genotype were chosen to be of the same age, harvested at the same time.

**Figure 1:**
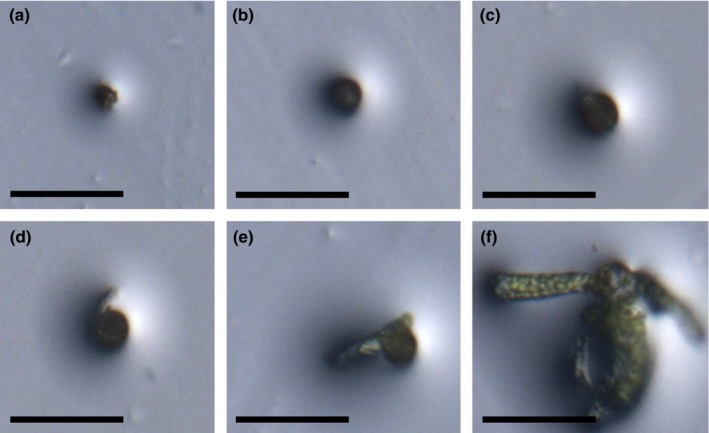
*Physcomitrella patens* spore germination stages. (a) Dry spore. (b) Imbibed spore. (c) Spore coat cracking. (d) Protrusion of one protonemal (chloronemal) filament. (e) Protrusion of two protonemal filaments. (f) Established protonemal colony. Bars, 100 μm.

Sporophytes were bleached in groups of two to three in 1 ml 25% Parozone^™^ (Jeyes Group, Thetford, UK) for 10 min and then washed three times in 1 ml sterile distilled water (10 min each) in a sterile flow cabinet. The sporophytes were then crushed in 100–200 μl of sterile water to release the spores. Spores were diluted down in sufficient sterile distilled water to allow plating of 500 μl of spore solution per Petri dish. Spores were plated on cellophane‐overlaid BCD supplemented with 5 mM CaCl_2_ and 5 mM ammonium tartrate, or on water agarose for the dormancy experiments in Fig. 1. Cellophane discs (A.A. Packaging Ltd, Preston, UK) were autoclaved for 15 min at 121°C, before use.

For hormone/chemical treatments, the treatment in the relevant solvent (or a solvent‐only control) was added to 1 ml of sterile water, which was then added to BCD medium just before plate‐pouring to achieve the desired final concentration in the plates. Within each assay, all solvent‐containing plates were matched so that all contained exactly the same volume of solvent, even if the hormone concentration varied. The solvents used were methanol for diterpenoids and norflurazon, acetone for GR24 and water for ethylene.

Plated spores were air‐dried in a laminar flow hood, sealed with micropore tape, and placed at 22°C in long‐day conditions. Spores were counted daily under the ×4 objective of a Leica compound microscope with a ×10 eyepiece; total magnification ×40. A minimum of 200 spores per plate was counted to define the number of germinated and ungerminated spores. Data were expressed as percentage germination, that is: (germinated spores/total spores counted) × 100. A spore was defined as having germinated as soon as the very first deformation of the spore coat by the emerging protonemal filament was observed.

Two to three technical replicates were contained within each assay, and each assay was repeated a minimum of three times on different dates, using different batches of spores and medium, to provide biological replicates. Data from one representative assay is shown in each figure.

### RNA preparation

RNA was prepared from dry spores (*c*. 250 harvested sporophytes), imbibed spores (*c*. 250 sporophytes bleached then soaked in liquid BCD for 18 h), germinating spores (spores from *c*. 250 bleached sporophytes plated at high density and left for 7 d until *c*. 50% germination was seen) and 100 mg vegetative (protonemal or leafy) tissue using the Bioline Isolate II Plant RNA preparation kit according to the manufacturer's instructions (London, UK). Average RNA yield was *c*. 300 ng μl^−1^, with OD260/280 > 2 and average OD 260/230 *c*. 1.8.

### RT‐PCR

Reverse transcription polymerase chain reaction (RT‐PCR) was carried out on 20 ng RNA from each sample using the Bioline MyTaq^™^ one step RT‐PCR kit according to the manufacturer's instructions. Primer sequences are detailed in Supporting Information Table S1.

### Generation of *Physcomitrella patens* lines with disrupted *PpCPS/KS* functionality

The moss line pCL755#29 is described in Zhan *et al*. (2015), generated using the methods described in (Bach *et al*., 2014). Briefly, a cassette containing *p35S‐nptII‐CaMVter*, expressing *Neomycin Phosphotransferase II* (conferring resistance to G418), was excised from pMBL6 (http://www.biology.wustl.edu/moss/pmbl6.jpg) using *Xho*I and inserted into the *Xho*I site in pDONR201:CPS/KS, generating the knock‐out construct pDONR201:CPS/KS‐nptII. Following transformation into *P. patens* of this construct, one line – pCL755#29 – with disrupted *PpCPS/K*S functionality was used for further studies.

A second line, pBK3, was generated by disrupting *PpCPS/KS* via targeted gene replacement using the pBK3 vector first described in Pan *et al*. (2015) and utilizing the method described in King *et al*. (2016), which contains a *p35S‐aph4‐CaMVter* cassette flanked by 5′‐ and 3′‐genomic sequence of *PpCPS/KS* on its 5′ and 3′ end, respectively. Therefore, genomic sequence of *PpCPS/KS* was replaced with *p35S‐aph4‐CaMVter*, which gives hygromycin resistance. The two lines were genotyped by PCR with the combination of primers that specifically bind to genomic DNA or selection marker cassette to distinguish knock‐out mosses from WT (Fig. S1).

### GC‐MS analysis of diterpenoids

All GC‐MS analyses were performed on a Shimadzu GCMS‐QP2010 plus (GC‐2010) with a CTC auto sampler AOC‐5000, with cooled trays, agitation oven, and needle bake‐out.

GC‐MS analysis utilizing solid‐phase microextraction fibers was previously published (Drew *et al*., 2012; Andersen *et al*., 2015). Briefly, the injection port temperature was set to 230°C, with a sampling time of 1 min. The flow control mode was pressure control with a total flow of 2.3 ml min^−1^, with H_2_ as carrier gas, and a purge flow of 1.0 ml min^−1^. The column was a 30 m HP‐5MS column. The oven temperature program was 35°C for 3 min, rising by 10°C per min to 230°C and a hold for 3 min. The MS settings were: Ion source temperature 260°C, interface temperature 280°C and the scan range from *m*/*z* 50 to *m*/*z* 350 with 70 eV electrical ionization.

All data were analysed using the Shimadzu software Lab Solutions, GCMS solutions v.2.70, using the libraries provided by NIST (NIST 08) and wiley (wiley 8.0). Obtained spectra were compared with the spectra in the mass spectral libraries. Compounds were identified comparing the data with library information of MS and retention indices (*I*). All reference *I*s were taken from Adams (2007).

## Results

### *Physcomitrella* spores are nondormant and do not require cold‐stratification or after‐ripening treatment for germination

**Figure 2:**
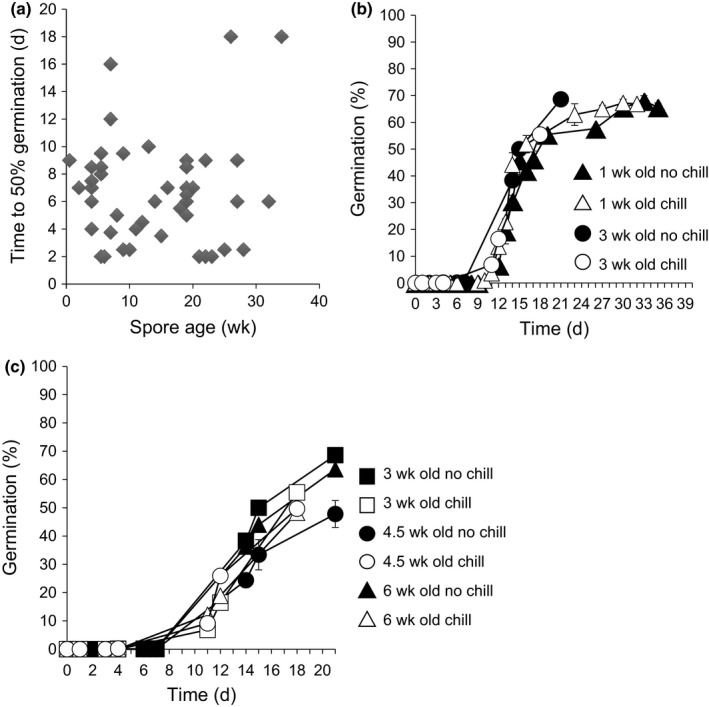
*Physcomitrella* spores do not appear to show primary dormancy and after‐ripening. (a) There is no correlation between dry spore age and time to 50% germination. Pearson–Spearman correlation test, *R* ^2^ = 0.004. (b) There is no effect of ageing or chilling on a single spore population. Spores from more than one sporophyte harvested from WT plants on the same day were germinated on water agarose with or without 3 d chilling at 4°C, 1 wk after collection and again at 3 wk after collection. Chilling does not accelerate germination. Error bars, ± SEM. (c) There is no effect of chilling on three independent batches of spores. Spores (from more than one sporophyte) from three independent harvests of different ages were germinated on water agarose with or without 3 d chilling at 4°C. Chilling does not accelerate germination. Error bars, ± SEM.

We define a *Physcomitrella* imbibed spore as having ‘germinated’ as soon as the first visible deformation of the spore coat occurs and the spore no longer appears spherical. This marks the first protrusion of protonemal filament(s), which grow with the subsequent formation of further filaments (Fig. 1a–f). Spores typically begin to germinate 2–7 d after imbibition/plating. We found that there was no correlation between spore age (time of dry storage post‐harvest) and speed of germination (Fig. 2a), suggesting that after‐ripening does not occur as it does in seeds. Moreover, a period of chilling, which breaks dormancy in seeds, did not affect spore germination (Fig. 2b,c). This suggests an absence of primary dormancy or after‐ripening in *Physcomitrella* spores.

### Far‐red inhibition of *Physcomitrella* spore germination is not rescued by application of diterpenes or inhibition of ABA synthesis

Although *Physcomitrella* spores appear not to have primary dormancy, we investigated whether their germination could be inhibited by environmental signals, as occurs in seeds (imposition of secondary dormancy in otherwise germination‐competent seeds). In both seeds and *Physcomitrella* spores, germination can be fully inhibited by a pulse of FR light (Seo *et al*., 2009; Possart & Hiltbrunner, 2013). In eudicot seeds, FR‐inhibition of germination can be fully rescued by treatment with bioactive GAs or by inhibition of ABA biosynthesis (Ikuma & Thimann, 1960; Schopfer *et al*., 2001; Oh *et al*., 2006; Seo *et al*., 2006; Lee *et al*., 2012).

**Figure 3:**
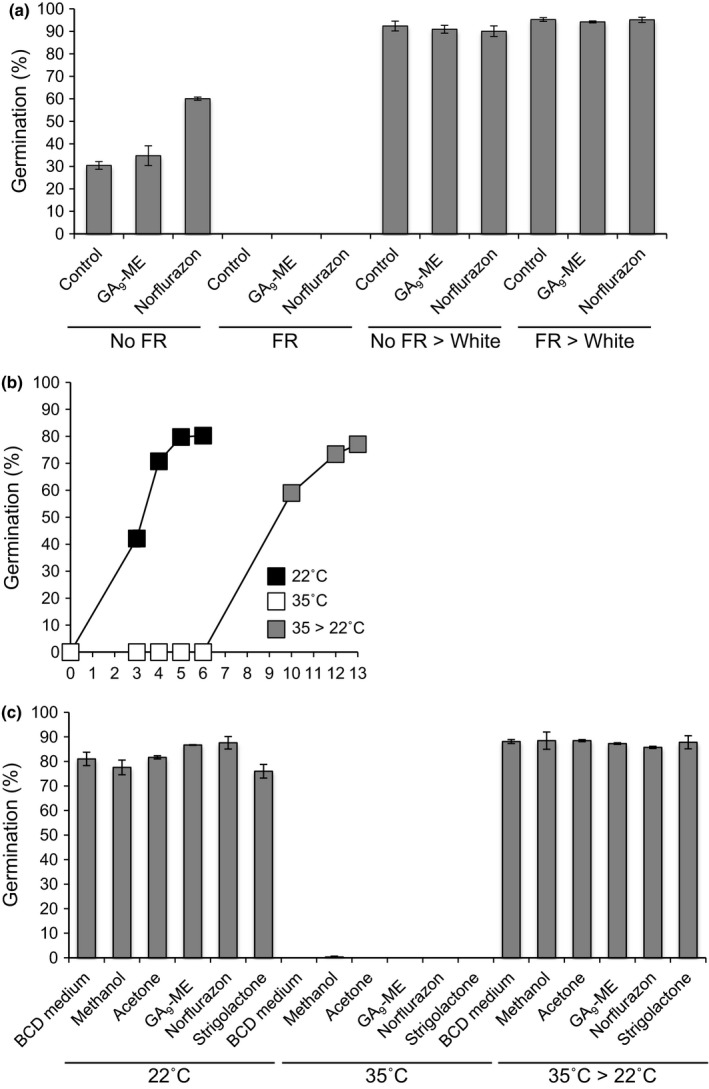
Effects of environmental signals on *Physcomitrella* spore germination. (a) A pulse of far‐red (FR) light inhibits spore germination and this cannot be rescued by norflurazon or GA_9_‐methyl ester (GA, gibberellin). Spores plated on control medium or GA_9_‐methyl ester (GA_9_‐ME) or norflurazon were treated ± FR light (‘No FR’ and ‘FR’, respectively) and immediately placed in the dark for 7 d. The spores’ germination percentage was measured 7 d after treatment. All spores were then moved to white light for a further 7 d (‘No FR > White’ and ‘FR > White’, under which conditions all spores germinated to completion, showing that the effect of FR light is completely reversible. A Kruskal–Wallis test indicates differences between FR and white light‐treated samples, *P *<* *0.05. Error bars, ± SEM. (b) A temperature of 35°C inhibits spore germination and this is completely reversible when spores are returned to 22°C. (c) High‐temperature inhibition of spore germination cannot be rescued by norflurazon, GA_9_‐methyl ester or the synthetic strigolactone analogue GR24 (‘strigolactone’ on the graph). Spores were germinated for 7 d at either 22°C or 35°C. The 35°C‐treated spores were then transferred to 22°C for another 7 d (‘35°C > 22°C’). A Kruskal–Wallis test indicated differences between 35°C and 22°C spores, *P *<* *0.05. Error bars, ± SEM.

In order to investigate whether the interface between light‐ and diterpene/ABA‐signalling in *Physcomitrella* spores is conserved with the regulation seen in *Arabidopsis* seeds, we FR‐treated spores in the presence of a diterpenoid known to be bioactive in moss (Hayashi *et al*., 2010), the fern antheridiogen GA_9_‐methyl ester, and the carotenoid biosynthesis inhibitor norflurazon, which blocks ABA biosynthesis in flowering plants (Chamovitz *et al*., 1991). We found that neither GA_9_‐methyl ester nor norflurazon were able to rescue the germination‐inhibitory effects of a pulse of FR light on *Physcomitrella* spores, even when control spores had germinated to 100% (Fig. 3a). This suggests that diterpenes and ABA do not facilitate spores’ responses to the environmental signal of light at certain wavelengths, and thus the hormonal control of *Physcomitrella* spores and dicot seeds is not conserved.

### *Physcomitrella* spore germination can be inhibited by high temperatures, but this cannot be rescued by ABA‐inhibition, or application of diterpenes or strigolactones

In order to further explore the role of diterpenes and ABA in the control of environmentally regulated germination, we inhibited spore germination using another environmental trigger: high temperature (thermoinhibition). Substantial and reversible thermoinhibition of seed germination (imposition of secondary dormancy) is seen at 32°C in *Arabidopsis* (Tamura *et al*., 2006; Toh *et al*., 2008), and this can be rescued by GA_3_, norflurazon or strigolactone application (Toh *et al*., 2012).

Incubation of spores at 35°C (but not 32 or 34°C; data not shown) caused complete inhibition of germination that was fully reversible upon return to normal growth conditions (22°C) (Fig. 3b). Thermoinhibition of *Physcomitrella* spores at 35°C could not be alleviated at all by GA_9_‐methyl ester, norflurazon or the synthetic strigolactone GR24 (Fig. 3c). Although high temperatures can inhibit germination in *Physcomitrella* spores, as in *Arabidopsis* seeds, the hormones mediating this response in *Arabidopsis* are not the same as in *Physcomitrella*, as was also seen with the FR light response.

### Diterpenoids can promote *Physcomitrella* spore germination

**Figure 4:**
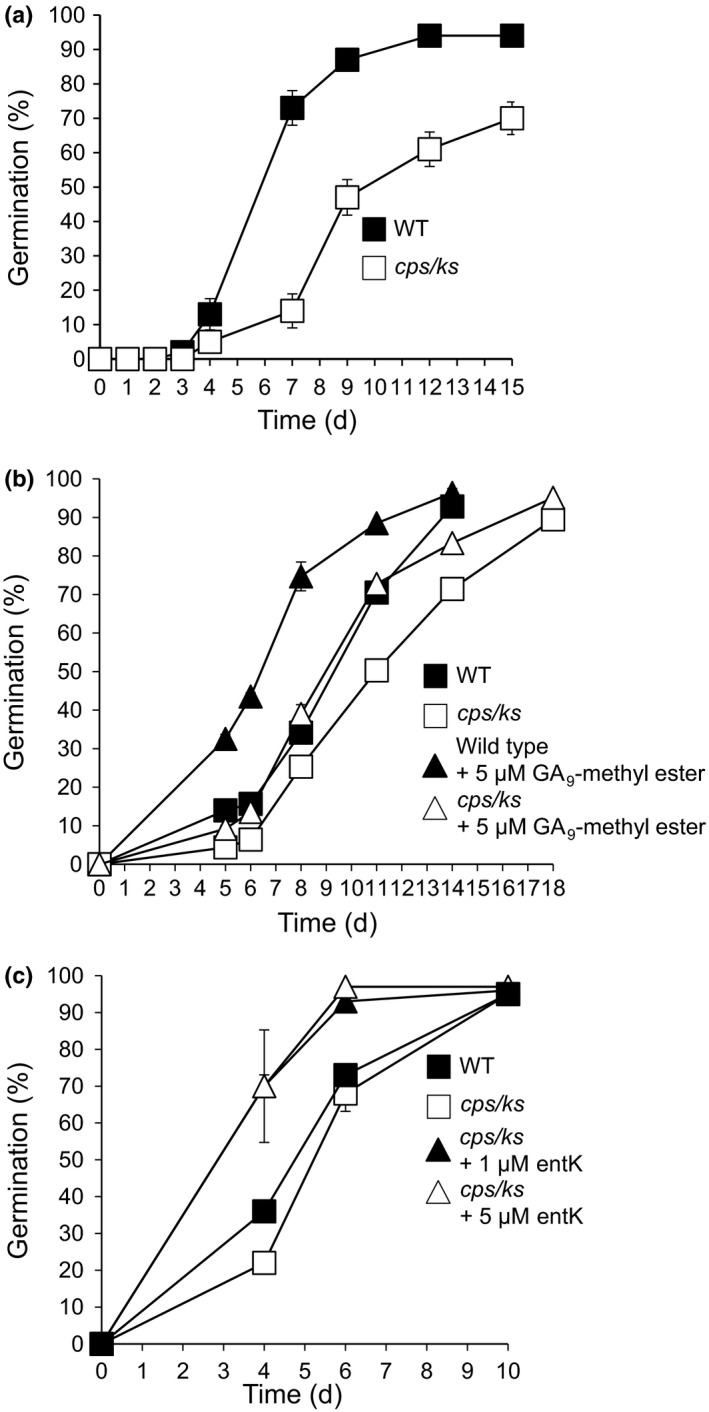
Diterpenes promote *Physcomitrella* spore germination. (a) Comparison of wild‐type (WT) and *copalyl‐diphosphate synthase/kaurene synthase* (*cps/ks*) mutant (Zhan *et al*., 2015) spore germination. (b) Effect of exogenous GA_9_‐methyl ester on WT and *cps/ks* mutant spores (GA, gibberellin). (c) Effect of exogenous *ent*‐kaurene (entK) on *cps/ks* mutant spores. Error bars, ± SEM.

Our previous work suggested conservation of some hormone function in spore and seed germination, via an ABA‐ARABIDILLO/PHYSCODILLO signalling module (Moody *et al*., 2016). Thus, we further explored the effects of diterpene hormones in *Physcomitrella*, to compare their effects with those of GAs in seeds. We examined *Physcomitrella* mutants in the gene encoding the first enzyme in the putative moss gibberellin biosynthesis pathway, *ent*‐*COPALYL DIPHOSPHATE SYNTHASE/ent*‐*KAURENE SYNTHASE* (*CPS/KS*), which makes no *ent*‐kaurene and hence no bioactive diterpenoids/gibberellins (*Ppcps/ks*; Hayashi *et al*., 2010; Fig. S1), similar to the mutants used by Hayashi *et al*. (2010). Two different *Ppcps/ks* mutant alleles showed a reduced germination speed compared with WT: they attained a lower percentage of germination at any given time on the upwards slope of the graph, although they eventually attained 100% germination (Figs 4a, S1a), and this phenotype could be rescued by application of two diterpenoids known to be bioactive in moss (Hayashi *et al*., 2010): the fern antheridiogen GA_9_‐methyl ester or *ent*‐kaurene (Fig. 4b,c). This result indicates that bioactive diterpenoid hormones in *Physcomitrella* have a positive effect on spore germination.

In order to further investigate this possibility, we examined the effect of diterpenes on WT *Physcomitrella* spore germination. GA_9_‐methyl ester and *ent*‐kaurene both enhanced spore germination (Figs 4b,c, S1b), whereas GA_3_ did not (Fig. S1c). Conversely, the diterpenoid hormones that promote moss spore germination cannot fully rescue the germination defect of the *Arabidopsis ga1‐3* mutant (Fig. S2). Together, these data: (i) show that diterpenoid hormones are not absolutely required for spore germination, unlike in seeds, corroborating Hayashi *et al*. (2010); (ii) indicate that diterpenoid hormones increase *Physcomitrella* spore germination speed, thus have a positive effect on germination; and (iii) lend support to the notion that bryophyte bioactive diterpenoid hormones differ from those in seed plants.

### ABA reduces *Physcomitrella* spore germination acting synergistically with sucrose

We have shown previously that ABA inhibits *Physcomitrella* spore germination in a dose‐dependent manner (Moody *et al*., 2016). *Physcomitrella* spores require approximately five‐fold higher concentrations of ABA for strong inhibition of germination than *Arabidopsis* (Finkelstein, 1994).

**Figure 5:**
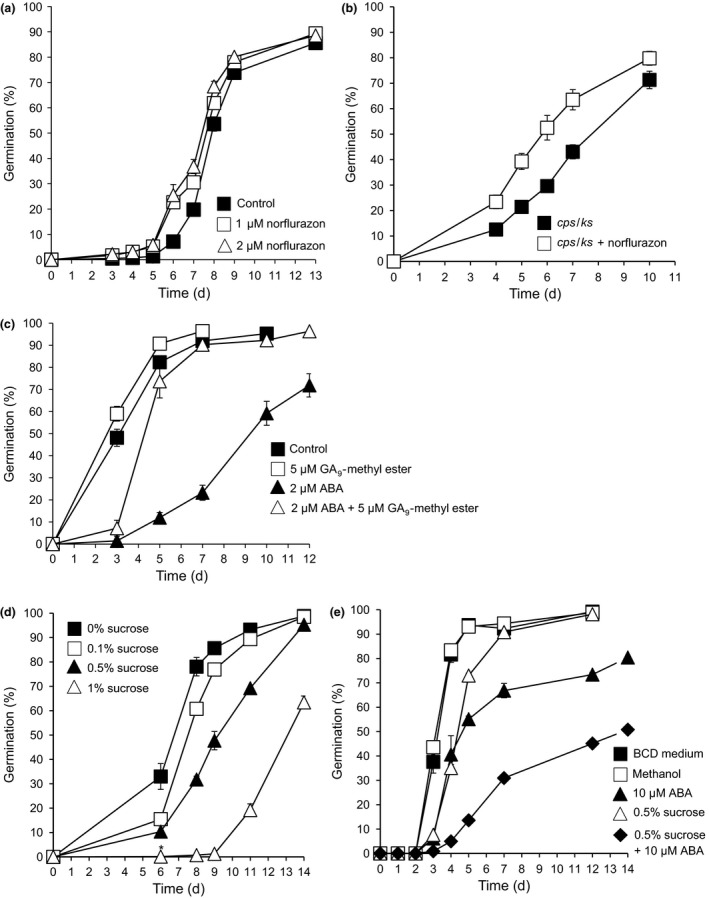
Abscisic acid (ABA) and sucrose synergistically inhibit *Physcomitrella* spore germination. (a) Effect of norflurazon on wild‐type (WT) spore germination. Five micromolar norflurazon has no further effect (data not shown). (b) Effect of 2 μM norflurazon on *cps* spores. (c) GA_9_‐methyl ester can rescue the inhibition of germination by ABA (GA, gibberellin). (d) Dose‐dependent inhibition of germination by sucrose. Sucrose was dissolved in BCD medium. (e) Synergistic inhibitory effect of ABA and sucrose on spore germination: an intermediate concentration of both ABA and sucrose was used; the 0.5% sucrose control also contains matched solvent. Error bars, ± SEM.

In order to examine the effect of inhibiting ABA biosynthesis on spore germination, we treated WT spores with norflurazon. We saw a small but reproducible promotion of germination (Fig. 5a; see also Fig. 3a). To investigate a potential link between diterpenoid hormones and ABA‐regulation of spore germination, we first tested whether the *cps/ks* mutant phenotype can be rescued via inhibition of ABA biosynthesis in moss spores. Norflurazon enhanced the germination of *cps/ks* mutant spores (Fig. 5b). Moreover, exogenously applied diterpenoid hormones could reverse the inhibitory effect of ABA on spore germination (Fig. 5c). This suggests that the balance of ABA and diterpenoid hormone levels may be important for regulating spore germination, but not with the same prominent role that these hormones have assumed in seed germination.

As ABA‐mediated stress responses in *Physcomitrella* vegetative tissue involve accumulation of soluble sugars (Burch & Wilkinson, 2002; Nagao *et al*., 2006; Oldenhof *et al*., 2006; Bhyan *et al*., 2012; Erxleben *et al*., 2012), we tested the effect of low concentrations of sucrose (0.1–1%) on spore germination. Sucrose inhibited spore germination in a dose‐dependent manner (Fig. 5d) and acted synergistically with ABA (Fig. 5e). Together these data show that ABA at relatively high concentrations reduces moss spore germination, as it does (although not as strongly as in) in seed germination, and suggest that *Physcomitrella* ABA‐mediated inhibition of spore germination could share downstream mechanisms with ABA‐mediated desiccation and freezing tolerance responses in the *Physcomitrella* gametophyte.

### *Ent*‐kaurene and ABA biosynthesis and signalling genes are expressed in spores

**Figure 6:**
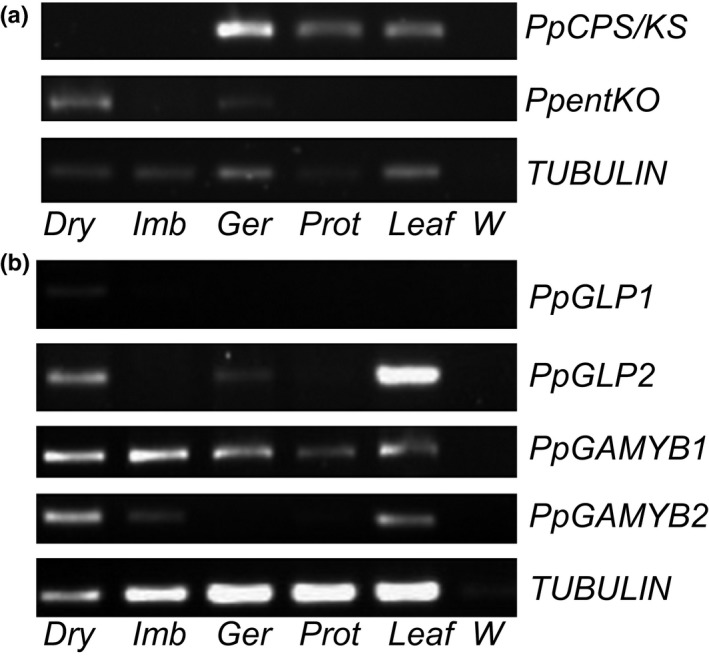
Expression of diterpene biosynthesis and putative response genes in *Physcomitrella* tissues. (a) Reverse transcription (RT)‐PCR of the *Physcomitrella* diterpene biosynthesis gene *PpCPS/KS* and putative diterpene biosynthesis gene *PpentKO* in *Physcomitrella* tissues compared to a *PpTUBULIN* control. (b) RT‐PCR of the putative *Physcomitrella* diterpene response genes *PpGLP1* and *PpGLP2* and *PpGAMYB1* and *PpGAMYB2* in *Physcomitrella* tissues compared to a *PpTUBULIN* control. Dry, Dry spores; Imb, imbibed spores; Ger, germinating spores; Prot, protonema; Leaf, Leafy gametophores; W, water control.

Our data suggest that diterpenoid hormones and ABA have subtle effects on spore germination compared with the absolute requirement for these hormones in regulating seed germination. To extend these findings, we asked whether the putative homologues of genes encoding the proteins responsible for biosynthesis and signal transduction of diterpenes and ABA are expressed in spores or during spore germination. We extracted RNA from dry spores, imbibed spores, germinating spores, protonemal filaments and leafy gametophytes. We performed semi‐quantitative RT‐PCR to detect expression of the *Physcomitrella* homologues of the *ent*‐kaurene biosynthesis genes *CPS*/*KS* and *CYP701A3* (*ent‐KO*) (Hayashi *et al*., 2006; Miyazaki *et al*., 2011), the first and second enzymes (respectively) in the putative moss diterpenoid hormone biosynthesis pathway (Hayashi *et al*., 2006). The *CPS/KS* transcript was detectable during spore germination and was absent from dry spores, whereas the *ent‐KO* transcript was detectable largely in dry spores (Fig. 6a), suggesting that spatial and temporal regulation of different stages of diterpene biosynthesis occurs during the *Physcomitrella* life cycle.

We also examined the expression of the putative gibberellin receptors *GLP1* and *GLP2* (Yasumura *et al*., 2007), and the two *PpGAMYB* transcription factors *PpGAMYB1* and *PpGAMYB2* (Aya *et al*., 2011). The putative gibberellin receptor *PpGLP1*, like *CYP701A3* (*ent‐KO*), showed its highest expression in dry spores, decreasing upon imbibition and undetectable once germination occurs (Fig. 6b). The second putative gibberellin receptor, *PpGLP2*, was expressed more strongly than *PpGLP1*, but again showed strong expression in dry spores, decreasing markedly during imbibition (Fig. 6b). *PpGLP2* expression also was detected in germinating spores and, later, in leafy tissue (Fig. 5b). *PpGAMYB1* was expressed in all tissues tested, whereas *PpGAMYB2*, similarly to the *PpGLP*s and *ent‐KO*, was detected in dry spores but decreased upon imbibition (Fig. 6b). *PpGAMYB2* was absent from germinating spores but present in protonemal and gametophyte tissues (Fig. 6b), corroborating Aya *et al*. (2011).

**Figure 7:**
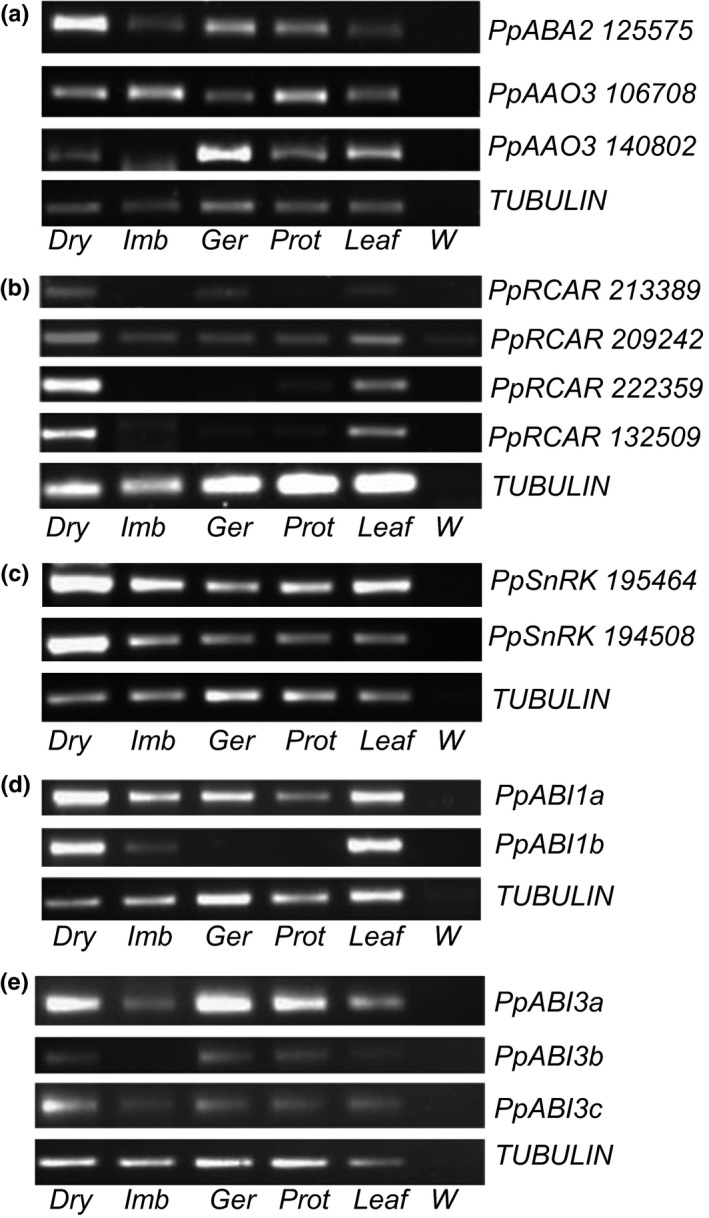
Expression of abscisic acid (ABA) biosynthesis and response genes in *Physcomitrella* tissues. (a) Reverse transcription (RT)‐PCR of the putative *Physcomitrella* ABA biosynthesis genes *PpABA2* and *PpAAO3* (two homologues; Hanada *et al*., 2011) in *Physcomitrella* tissues. (b) RT‐PCR of the four putative *Physcomitrella* ABA receptors (RCARs; Hanada *et al*., 2011) in *Physcomitrella* tissues. (c) RT‐PCR of the two putative *Physcomitrella* ABA signalling kinases (SnRKs; Hanada *et al*., 2011) in *Physcomitrella* tissues. (d) RT‐PCR of the *Physcomitrella ABI1* ABA‐response genes (Sakata *et al*., 2009) in *Physcomitrella* tissues. (e) RT‐PCR of the *Physcomitrella ABI3* ABA‐response genes (Khandelwal *et al*., 2010) in *Physcomitrella* tissues. All gene expression was compared to a *PpTUBULIN* control. Dry, dry spores; Imb, imbibed spores; Ger, germinating spores; Prot, protonema; Leaf, Leafy gametophores; W, water control.

We also assessed the expression of the putative genes encoding the final two (cytosolic, ABA‐specific) steps in the ABA biosynthesis pathway, namely two putative *Physcomitrella ABA DEFICIENT2* (*ABA2*) homologues and two putative *Physcomitrella ABSCISIC ALDEHYDE OXIDASE3* (*AAO3*) homologues (Hanada *et al*., 2011). All genes showed expression in dry spores, germinating spores and leafy tissue, with one *PpABA2* and one *PpAAO3* also present in imbibed spores and one *PpAAO3* also present in protonema (Fig. 7a). We also tested the expression of putative ABA signalling genes. The four putative *PYRABACTIN‐RESISTANCE 1/PYRABACTIN RESISTANCE 1‐RELATED/REGULATORY COMPONENT OF ABA RECEPTOR* (*PYR/PYL/RCAR*) ABA receptors (Takezawa *et al*., 2011) were expressed in all tissues tested (Fig. 7b), as were the two putative Class II SnRK phosphatases that were detectable in this assay (Fig. 7c) (out of the total six SnRKs in *Physcomitrella*; Takezawa *et al*., 2011). Out of the two *Physcomitrella ABI‐INSENSITIVE1* (*ABI1*) protein phosphatases (Komatsu *et al*., 2013), *PpABI1a* was expressed ubiquitously (Fig. 7d), whereas *PpABI1b* was highly expressed in dry spores and leafy tissue (Fig. 7d). Out of the three ABA‐regulated transcription factors *Physcomitrella ABA INSENSITIVE3A*, ‐*3B* and *‐3C* (*PpABI3A*,* ‐3B* and *‐3C*)(Khandelwal *et al*., 2010), *PpABI3A* and *PpABI3C* were expressed in all tissues tested, whereas *PpABI3b* was largely absent from imbibed spores but present in other tissues (Fig. 7e).

In summary, all putative ABA biosynthesis genes were expressed in dry spores, with lower levels during imbibition, as is the putative second gene in the diterpene biosynthesis pathway. The initial diterpene biosynthesis gene transcript, *PpCPS/KS*, was detected only after imbibition during germination and growth. Putative ABA signalling genes were largely expressed ubiquitously, although many were expressed more highly in dry spores than imbibed spores. Putative GA signalling genes (receptors and GAMYBs) were all expressed in dry spores but largely decreasee in expression level during imbibition.

### Strigolactones inhibit *Physcomitrella* spore germination

**Figure 8:**
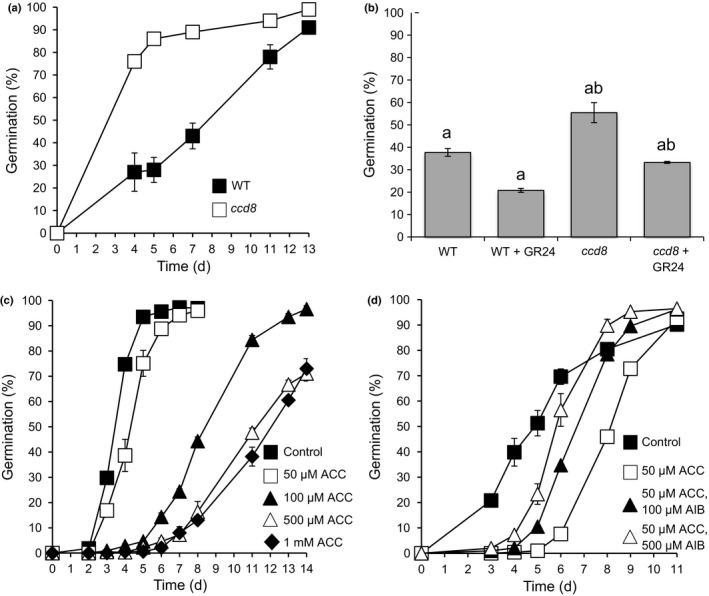
Strigolactones and an ethylene precursor reduce *Physcomitrella* spore germination rate. (a) *Ppccd8* strigolactone biosynthesis mutants (Proust *et al*., 2011) germinate faster than their corresponding wild‐type (WT). (b) *Ppccd8* mutant germination can be inhibited by exogenously applied synthetic strigolactone GR24, which also inhibits WT spore germination. Day 5 data shown; a Kruskal–Wallis test indicates significant differences between samples, *P *<* *0.05 as indicated by the letters on the graph. (c) The ethylene precursor 1‐aminocyclopropane‐1‐carboxylic acid (ACC) inhibits WT spore germination rate. (d) The ethylene inhibitor aminoisobutyric acid (AIB) can rescue the effects of ACC on spore germination. Error bars ± SEM.

In order to extend our findings around the hormonal control of spore germination, we examined the effect of other hormones known to affect seed germination. Strigolactones promote seed germination in a variety of plants (Akiyama & Hayashi, 2006) and have been suggested to affect *Physcomitrella* spore germination (Proust *et al*., 2011). We tested whether strigolactones affected spore germination by comparing the germination of WT spores with those of the *Physcomitrella ccd8* mutant, which cannot synthesize SLs (Proust *et al*., 2011). The *ccd8* mutant showed increased germination (Fig. 8a), corroborating an unpublished observation by Proust *et al*. (2011). The *ccd8* mutant's increased germination could be reduced to levels closer to that of WT spores by exogenous application of GR24 (Fig. 8b). Moreover, exogenous GR24 reduced the germination speed of WT *Physcomitrella* spores at concentrations of 0.1 μM (Fig. 8b). This indicates that in *Physcomitrella*, unlike in *Arabidopsis* and parasitic plants, strigolactones have an inhibitory role in the germination process.

### The ethylene precursor ACC inhibits *Physcomitrella* spore germination

Like strigolactone, ethylene has been shown to promote seed germination in *Arabidopsis* and other dicots, acting antagonistically to ABA. We showed that application of 50 μM–1 mM 1‐aminocyclopropane‐1‐carboxylic acid (ACC), the ethylene precursor that is cleaved by ACC oxidase to release ethylene, inhibited *Physcomitrella* spore germination in a dose‐dependent manner (Fig. 8c), which is the converse of its effects in seeds (where experiments commonly use a dose of 1 mM ACC), but similar to strigolactone's effects on spores. Moreover, the ethylene biosynthesis inhibitor aminoisobutyric acid, which competitively inhibits ACC oxidase, can overcome the effects of ACC (Fig. 8d).

## Discussion

### Environmental regulation of germination by light and high temperature is not integrated by the same hormones in *Physcomitrella* spores compared with seeds

We have shown that unlike most seeds, *Physcomitrella* spores grown in laboratory conditions do not show primary dormancy and after‐ripening. A lack of primary dormancy has been reported in several moss and liverwort species (McLetchie, 1999; Glime, 2015).

We have also shown that, in a similar way to seeds, germination‐competent *Physcomitrella* spores can be prevented from germinating using environmental cues. This is in line with evidence that conditional (secondary) dormancy exists in *Sphagnum* allowing formation of a spore bank (Sundberg & Rydin, 2000) and in *Physcomitrium sphaericum* (Furness & Hall, 1981). The interface of environmental signals with known hormonal networks is not conserved between *Physcomitrella* spores and flowering plant seeds.

Seed germination and spore germination can both be reversibly inhibited by a pulse of far‐red (FR) light (e.g. Seo *et al*., 2006; Possart & Hiltbrunner, 2013) or elevated temperatures (32°C in *Arabidopsis*, Toh *et al*., 2008; 35°C in *Physcomitrella*, this work). This shows that both spores and nondormant seeds respond to environmental cues that block germination, and suggests that this is an evolutionarily early adaptation for plants to live, and move around, on land. Our experiments suggest a higher degree of temperature tolerance in *Physcomitrella* spores compared with seeds: *Physcomitrella* vegetative tissue is also more tolerant to abiotic stress than that of seed plants (Frank *et al*., 2005). Temperatures of 35°C are known to inhibit germination in the moss *Physcomitrium* (Furness & Hall, 1981) and in two species of liverwort (Chopra & Kumra, 1988), although this was not shown to be reversible (Chopra & Kumra, 1988).

Unlike in seeds (Ikuma & Thimann, 1960; Schopfer *et al*., 2001; Oh *et al*., 2006; Seo *et al*., 2006; Nelson *et al*., 2009), FR‐inhibition of *Physcomitrella* spore germination cannot be rescued by addition of diterpenes or inhibition of abscisic acid (ABA) synthesis. Furthermore, unlike in seeds (Toh *et al*., 2012), addition of diterpenes, norflurazon or strigolactones (SLs) cannot rescue the germination of spores inhibited by high temperature. Thus, a rescue mechanism for FR‐ and thermo‐inhibition in *Physcomitrella* spores is divergent compared with seeds, and remains to be elucidated.

In seeds, environmentally regulated inhibition of germination impinges on ABA and gibberellin (GA) synthesis and metabolism (Seo *et al*., 2006; Toh *et al*., 2012). Crosstalk between light and GA/ABA signalling in seeds occurs at multiple levels, including via effects on hormone metabolism (and subsequent hormone levels), via transcriptional changes in signalling genes (reviewed in Piskurewicz *et al*., 2009), and via interactions between transcription factors from different pathways (e.g. Richter *et al*., 2010; Casal, 2013; Tang *et al*., 2013). For example, FR light blocks germination in dicots by repressing the expression of *GA*_*3*_ *‐oxidase* (Toyomasu *et al*., 1998; Yamauchi *et al*., 2004), which catalyses a step in gibberellin biosynthesis that does not exist in *Physcomitrella* (Stewart & Freebairn, 1969; Hayashi *et al*., 2010; Zhan *et al*., 2015). Moreover, *Physcomitrella* seems not to have clear orthologues of FHY3/FAR1 or ABI5 (Rensing *et al*., 2008) (which integrate ABA and light signalling in *Arabidopsis*; Tang *et al*., 2013) and has divergent DELLA proteins that have not been shown to transduce gibberellin signalling (Yasumura *et al*., 2007). Thus, the ‘wiring’ of the interface between environmental and hormonal regulation of seed germination evolved after the divergence of the bryophyte lineage. Our work suggests that a novel trigger for light‐induced germination exists in moss. Furthermore, divergent molecular networks mediate conserved developmental responses to environmental stimuli in spores and seeds to enable plant movement on land.

### Diterpenes and ABA affect germination in *Physcomitrella* spores but appear to have a modulatory role, in contrast to the critical role of GA and ABA in seeds

We showed, on the one hand, that certain diterpenoid hormones have a positive effect on germination in *Physcomitrella* spores and, on the other, that ABA has a negative effect on spore germination. Furthermore, *Physcomitrella ent*‐*KO*, ABA synthesis genes and putative diterpene‐ and ABA‐signal transduction pathway genes were expressed in spores. Corroborating previous work (Hayashi *et al*., 2010; Zhan *et al*., 2015), we saw that the bioactive diterpenes in *Physcomitrella* are those at an early step in the biosynthesis pathway (*ent*‐kaurene), or those that show activity in ferns and also spore‐bearing plants (GA_9_‐methyl ester), rather than those active in seed plants. We found that diterpenoids are not required for *Physcomitrella* spore germination (corroborating Hayashi *et al*., 2010), but that they do influence germination in a positive way. Although Hayashi *et al*. (2010) did not report a germination phenotype for their *cps* mutant, their mutant spores were compared with wild‐type (WT) only at a single time point, so no measure of germination rate was made and thus relatively subtle differences were probably overlooked. *Physcomitrella* GAMYB proteins, homologues of which are regulated by gibberellin signalling in flowering plants, are required for correct spore coat formation (Aya *et al*., 2011).

Mutant analysis in *Arabidopsis* has demonstrated that bioactive gibberellins and gibberellin signalling are absolutely required for seed germination to occur (Koornneef & van der Veen, 1980), whereas *Arabidopsis* seed germination is completely inhibited by concentrations of ABA as low as 5 μM (Finkelstein, 1994). The effects of gibberellins and ABA in *Physcomitrella* spores were not as extreme: the *cps* mutant has a slower germination rate than WT, but *cps* mutant spores can eventually germinate to the same level as WT controls, demonstrating no loss of germination potential. The concentration of ABA required for strong inhibition of *Physcomitrella* spore germination was also five‐ to 10‐fold higher than for *Arabidopsis* seeds, although different spore batches vary in their ABA sensitivity, as is the case for *Arabidopsis* seeds (Finkelstein, 1994), as might be expected in an ephemeral species (Glime, 2013).

The effect of norflurazon on *Physcomitrella* spores was subtle, which may be due to the lack of primary dormancy in spores, which are essentially ‘ready to germinate’. The extent to which norflurazon reduces ABA levels in *Physcomitrella* is not known.

This suggests that diterpenes and ABA perform a modulatory role during *Physcomitrella* spore germination, and gained a more prominent and complex role in multicellular seeds after co‐option into the sporophyte during the evolution of seed plants (e.g. Piskurewicz *et al*., 2009). Our data comparing the behaviour of *physcodillo* mutant spores and *arabidillo* mutant seeds on ABA suggest that these Armadillo‐related protein homologues may represent a conserved node in an ancient regulatory network (Moody *et al*., 2016).

The function of ABA in *Physcomitrella* previously has been studied in gametophytic vegetative tissues, where a role in stress responses (such as drought) has been demonstrated, as in the flowering plant sporophyte (Knight *et al*., 1995; Cuming *et al*., 2007; Khandelwal *et al*., 2010; Takezawa *et al*., 2011). Several studies have demonstrated a role for sugars (including sucrose) acting synergistically with ABA during moss abiotic stress resistance (Burch & Wilkinson, 2002; Nagao *et al*., 2006; Oldenhof *et al*., 2006; Bhyan *et al*., 2012; Erxleben *et al*., 2012). We demonstrated that a similar synergism may exist during spore germination. The level of sucrose that can decrease spore germination rate (0.1–1% in this study) is 10‐ to 100‐fold lower than that used to cause osmotic stress in moss (10%: Garrocho‐Villegas & Arredondo‐Peter, 2008). Evidence exists for a regulatory role of sugars during seed germination: an inhibitory effect of low levels of sucrose (0.5–3%) on *Arabidopsis* seed germination up to 5 d has been observed (Chen *et al*., 2006; Li *et al*., 2012). This effect requires functional ABA biosynthesis, because *aba2* mutant seeds are sucrose‐insensitive (Li *et al*., 2012). However, Finkelstein & Lynch (2000) showed that 1–2% sucrose could counteract the effects of 3 μM ABA on *Arabidopsis* seed germination after 7 d, suggesting that different interactions between sucrose and ABA may occur at different times.

### A novel role for SLs and ethylene in inhibiting spore germination in *Physcomitrella*

We showed that SLs have a negative effect on *Physcomitrella* spore germination. The role of SLs in seed plant germination is a positive one: they act as signals to promote seed germination either between plants (via root exudates) or within one plant (e.g. during thermoinhibition) (Bouwmeester *et al*., 2003; Yoneyama *et al*., 2010; Toh *et al*., 2012; Stanga *et al*., 2013). This occurs via regulation of gibberellin biosynthesis (Nelson *et al*., 2009), lending weight to the hypothesis that SLs have a different mechanism of action in *Physcomitrella* germination. SLs are well known as regulators of shoot branching in seed plants (Waldie *et al*., 2014). This branching function is conserved in the *Physcomitrella* gametophyte (Proust *et al*., 2011). In addition, SLs in moss promote ‘self‐awareness’ and delimit colony spread, acting as quorum‐sensing molecules (Proust *et al*., 2011), suggesting that the ‘between‐plant’ communication function of SLs arose early in land plant evolution. Perhaps in bryophytes the inhibitory role of SLs in spore germination arose as a quorum‐sensing function; thus, when spores are released from the plant, the formation of one colony from a spore could prevent the germination of a second colony‐forming spore close by, and hence aid colony establishment without competition for resources.

Interestingly, ethylene also has a positive role in seed germination and the ethylene precursor ACC has a negative role in spore germination: as ethylene is also a small, gaseous and easily diffusible hormone, its presence in a developing moss gametophyte could also signal to neighbouring spores and prevent their germination. Ethylene affects seed germination via crosstalk with ABA signalling/synthesis, via synergism with GA signalling and via direct effects on cell separation of the endosperm in a number of plant species (Linkies & Leubner‐Metzger, 2012). This suggests that ethylene may have been co‐opted separately into spores and seeds to perform different roles.

### Comparing the regulation of germination in *Physcomitrella* spores and nondormant seeds

The regulation of equivalent developmental processes between gametophyte and sporophyte seems to show an amazing degree of similarity in comparative studies on key model organisms. For example, *Physcomitrella* rhizoids are developmentally equivalent to *Arabidopsis* root hairs (Menand *et al*., 2007), and GAMYB functions in spore and reproductive organ development are equivalent (Aya *et al*., 2011), whereas the liverwort *Marchantia polymorpha* shows circadian regulation of the vegetative‐to‐reproductive transition, as in the seed plant sporophyte (Kubota *et al*., 2014). We have shown that spores and seeds respond to the same environmental cues to generate the same developmental output, but via different mechanisms.

Our results suggest evolution of novel hormonal regulation of germination between *Physcomitrella* spores and nondormant/after‐ripened seeds, with ABA and GA assuming much greater importance in seed plants, multilevel crosstalk between environmental and hormone pathways evolving in seeds, and some hormones being co‐opted into different roles in spores and seeds. Similar network rewiring has been identified between root hairs and rhizoids, between RHD SIX‐LIKE transcription factors and auxin signalling (Jang *et al*., 2011; Pires *et al*., 2013).

One possibility is that the multicellular nature of seeds may have led to a requirement for complex, coordinated hormonal regulation of the different tissues during germination. Determining the molecular nature of the signalling pathways that regulate spore germination is now a key target for future research. Alternatively, as spores appear not to show dormancy, there may be no requirement for a complex hormonal regime regulating dispersal. It is possible that a ‘bet‐hedging’ strategy controlling germination rates due to life history may exist in *Physcomitrella* spores as in *Arabidopsis* (Springthorpe & Penfield, 2015). The absence of observed dormancy in moss spores also raises the question of how these dispersal units function to colonize new environments.

## Author contributions

E.F.V., Y.S., L.A.M., G.W.B., H.T.S. and J.C.C. planned and designed the research and conceived the experiments. E.F.V., Y.S., L.A.M., D.H., A.W., S.N., A.C., B.B., D.M., S.J.B., H.B., B.C.K. and J.C.C. performed the research. E.F.V., Y.S., L.A.M., D.H., A.W., S.N., H.B., B.C.K., G.W.B., H.T.S. and J.C.C. analysed data. E.F.V., L.A.M., H.B., G.W.B., H.T.S. and J.C.C. wrote the paper.

## Supporting information

Please note: Wiley Blackwell are not responsible for the content or functionality of any supporting information supplied by the authors. Any queries (other than missing material) should be directed to the *New Phytologist* Central Office.

**Fig. S1** Moss bioactive gibberellins promote *Physcomitrella* spore germination.
**Fig. S2** Gibberellins that are bioactive in *Physcomitrella* cannot rescue the *Arabidopsis ga1‐3* mutant seed germination phenotype and substitute for GA_3_.
**Table S1** Primers used for RT‐PCR analysis
