# Supplementary material for: The decision to germinate is regulated by divergent molecular networks in spores and seeds
Source: New Phytol. 2016 Jun 3;211(3):952–66. doi: 10.1111/nph.14018 (PMC4950004; doi:10.1111/nph.14018)
Supplement: Supplementary file 1 — Fig. S1 Moss bioactive gibberellins promote Physcomitrella spore germination. Fig. S2 Gibberellins that are bioactive in Physcomitrella cannot rescue the Arabidopsis ga1‐3 mutant seed germination phenotype and substitute for GA3. Table S1 Primers used for RT‐PCR analysis [file NPH-211-952-s001.pdf]

New Phytologist

***New Phytologist* Supporting Information Figs S1 & S2 and Table S1**

Article title: The decision to germinate is regulated by divergent molecular networks in spores and seeds

Authors: Eleanor F. Vesty, Younousse Saidi, Laura A. Moody, Daniel Holloway, Amy Whitbread, Sarah Needs, Anushree Choudhary, Bethany Burns, Daniel McLeod, Susan J. Bradshaw, Hansol Bae, Brian Christopher King, George W. Bassel, Henrik Toft Simonsen and Juliet C. Coates

Article acceptance date: 16 April 2016

The following Supporting Information is available for this article:

Fig. S1 Moss bioactive gibberellins promote *Physcomitrella* spore germination.

Fig. S2 Gibberellins that are bioactive in *Physcomitrella* cannot rescue the *Arabidopsis ga1-3* mutant seed germination phenotype and substitute for GA_3.

Table S1 Primers used for RT-PCR analysis

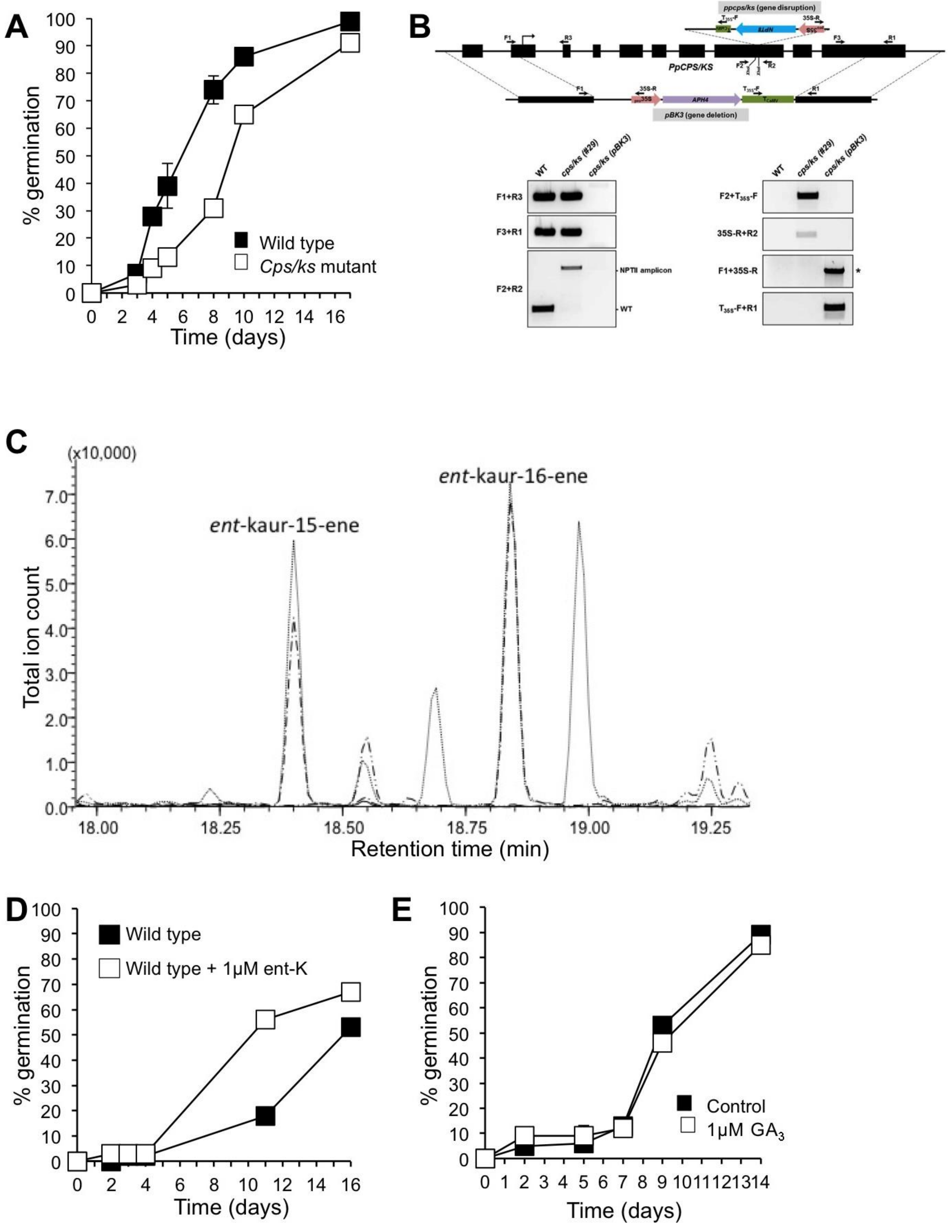

Fig. S1 Moss bioactive gibberellins promote *Physcomitrella* spore germination. (a) *Ppcps/ks*

mutant (pBK3 targeted gene replacement) shows slower germination than wild type. Error bars ± SEM. (b) Genotyping of pBK3 *cps/ks* gene replacement mutant compared to the *Ppcps/ks* disruption line 29 (Zhan *et al*., 2015) and wild type. (c) Lack of diterpenes in both *Ppcps/ks* mutants compared to wild type. The figure shows that the two major peaks produced by the CPS/KS enzyme, *ent*-kaur-15-ene and *ent*-kaur-16-ene, are not in either mutant. Narrow dotted line, wild type spores; alternate dashed and dotted line, wild type green tissue; alternate dashed and two-dotted line, *Ppcps/ks* pBK3; solid line, *Ppcps/ks* #29 gene disruption. The two additional major peaks in wild type spores are unknown, non-terpenoid compounds. (d) *ent*-kaurene promotes germination in wild-type spores. (e) GA_3 cannot promote germination in wild-type spores.

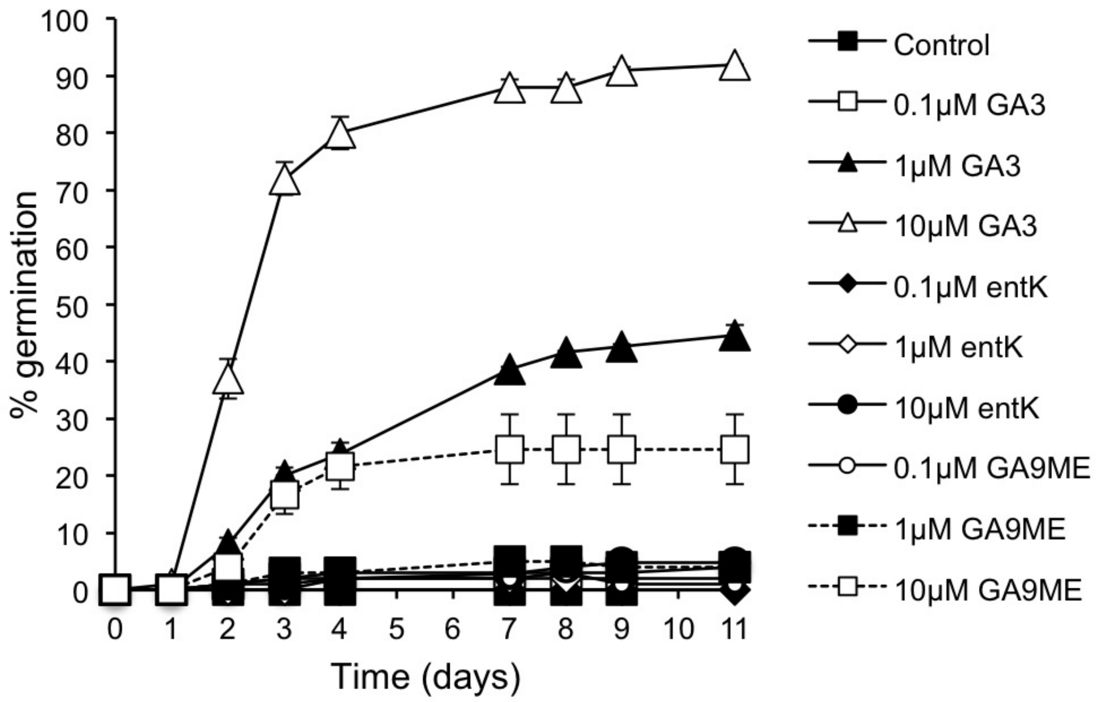

Fig. S2 Gibberellins that are bioactive in *Physcomitrella* cannot rescue the *Arabidopsis ga1-3* mutant seed germination phenotype and substitute for GA_3. *ga1-3* seeds were plated on 0.5 MS medium and germination (radicle emergence) was scored in the control (methanol solvent) and in seeds treated with different gibberellins (solvent-matched) as shown. Error bars ± SEM.

New Phytologist

Table S1 Primers used for RT-PCR analysis; the gene that the primer hybridizes to is indicated, along with whether it is a forward (fwd) or reverse (rvs) primer

Sequence	Name
AACAAGAGTTGCACATGGCGTA	PpDELLAa fwd
GGTGGCAGACTAAGCAGCTC	PpDELLAa rvs
CTGGAGAACAACGCGATGGC	PpDELLAb fwd
CCCTCGCTGGATAGATTCCG	PpDELLAb rvs
CTCTATTACCATGGAGGCGGA	GLP1 GID1-like fwd
CCGAATATCTCATCTCCCAATC	GLP1 GID1-like rvs
TTATTACTACCACGGAGGCGGG	GLP2 GID1-like fwd
CTCCGCAAATCGCATCTCCCAA	GLP2 GID1-like rvs
GGATGTATGGGTGCGTCTTTTC	GLP3 GID1-like fwd
TGCGTATCTCGTCTCCCAGTC	GLP3 GID1-like rvs
CTGCCCAGGCGAGCTCCGG	GLP4 GID1-like fwd
GAACATATGGACGCCATCCTCG	GLP4 GID1-like rvs
CCCAATCACTCCCGGGCCGT	GLP5 GID1-like fwd
AGCTCCGCGACTACGACCAGA	GLP5 GID1-like rvs
AATGCAGGCGGTGAGAGTCCC	GLP6 GID1-like fwd
GGGATCTTTGCCACCTACAA	GLP6 GID1-like rvs
CATGGCTGCCCAACTTCCCG	PpGAMYB1 fwd
GAGCGGACTAGGATTGGTAATC	PpGAMYB1 rvs
TTGATGCCTTAATGCAGGATGC	PpGAMYB2 fwd
GCGGAGCACACGGAACAGG	PpGAMYB2 rvs
CACAGACTTCCGATACCCATGG	CPS fwd
GCCTTGGCATCTTCCATCATCG	CPS rvs
CCTTCGCCTTGCAGCAGGTG	ent-kaurene oxidase fwd
AGCGCACATCCTCTTTCCAGC	ent-kaurene oxidase rvs
ATGGCTTCCAGCACCTTGATAC	PpCPS/KS fwd
TCAGGTACTGGCTCGAAGAGC	PpCPS/KS rvs
AGACCGTCCGGAGGTGACTG	PpABI1a fwd

GCGGACTCAACTTCCTCTGCT	PpABI1a rvs
TATGCCTGGTGACTTATACCAG	PpABI1b fwd
CTCGCTCTGGCTTGTGATCC	PpABI1b rvs
CGGTTGATGGTTGAGGGCGA	PpABI3A fwd
GGCCAAAACCTGTATCGAATGT	PpABI3A rvs
GAAATGAGAAAAGTCCCTGCCC	PpABI3b fwd
TCGGCCAGAACCTGTATCTGAT	PpABI3b rvs
CAGCAGCAGAGGCAGAGGCG	PpABI3C fwd
TGCGCCCCTTCTGTTCAGCA	PpABI3C rvs
TCAGAACTTGTTTGAGGGGATC	ABA2 Phypa125575 fwd
GCTGGCACAGTATGTGTGGG	ABA2 Phypa125575 rvs
CTACAGCATGTGGCATCTCCG	ABA2 Phypa202254 fwd
ACTCGAATACCGTAACCCGCAT	ABA2 Phypa202254 rvs
TCCTTTCCGGAGAGCCAACC	AAO3 Phypa106708 fwd
TGTCGTCACTATGCCCTCAGA	AAO3 Phypa106708 rvs
GGGCACGCACAATGTAACGTCA	AAO3 Phypa140802 fwd
GCACCTTCAACCTGTCCGACG	AAO3 Phypa140802 rvs
GAGAAACAGGGGCGGCCGGA	Phypa209242 fwd
GCCGCTGGTTTCTCGTTGGAG	Phypa209242 rvs
GATGCTACCCACCCCGCCA	Phypa222359 fwd
CCCCTCTCCATATCTAAGCATT	Phypa222359 rvs
CGTCACAGGGCAGCGGCG	Phypa132509 fwd
CAGGTGCAAATTACAGTACTGG	Phypa132509 rvs
AGGAGGAGCACGCGTACGCA	Phypa213389 fwd
GGACGCTGTGAGCACGCAAG	Phypa213389 rvs
GTGAAGGACATTGGGTCGGG	SnRK2 Phypa195464 fwd
CGGGATCCTCAAACGGATATG	SnRK2 Phypa195464 rvs
TTCGAAGTCCTCCTTGTTGCAC	SnRK2 Phypa194508 fwd
CCCACATCGGGATCCGCATC	SnRK2 Phypa194508 rvs
TCCTCCTGTACTTGCTTCTGG	SnRK2 Phypa106968 fwd
CGCTCAAGATACGTCCAATGG	SnRK2 Phypa106968 rvs

ACTCGGGAGCTTGTTGCGGTG	SnRK2 Phypa215231 fwd
GGACCCCAACCATCCCCCTC	SnRK2 Phypa215231 rvs
TGTGCTGTTGGACAATGAG	Pptub fwd
ACATCAGATCGAACTTGTG	Pptub rvs

References

Zhan X, Bach SS, Hansen NL, Lunde C, Simonsen HT. 2015. Additional diterpenes from *Physcomitrella patens* synthesized by copalyl diphosphate/kaurene synthase (PpCPS/KS). *Plant Physiol Biochem* **96:** 110–114.
